# Perceptual Effects of Walnut Volatiles on the Codling Moth

**DOI:** 10.3390/insects15060402

**Published:** 2024-05-30

**Authors:** Peixuan Li, Yang Wei, Guoxiang Chen, Adil Sattar

**Affiliations:** 1 College of Horticulture, Xinjiang Agricultural University, Urumqi 830052, China; hnlpx0078@163.com; 2 College of Forestry and Landscape Architecture, Xinjiang Agricultural University, Urumqi 830052, China; 15199946377@163.com (Y.W.); 18199892144@163.com (G.C.)

**Keywords:** *Cydia pomonella*, VOCs, olfactory response, EAG, GC-EAD

## Abstract

**Simple Summary:**

The codling moth, *Cydia pomonella* (L.), is a worldwide destructive borer in fruits. Walnuts, *Juglans regia* (L.), have suffered significant damage due to the infringement of this pest. Out of concern for ecological security, volatile organic compounds (VOCs) in plants are widely used in pest control. However, while a great deal of research has focused on apples and pears, the compounds in walnuts that attract codling moths have not been clearly defined. In this study, we used a field survey to identify the walnut organs in which the codling moths lay eggs and then we extracted volatiles from these walnut organs for analysis and evaluated the electrophysiological and behavioral responses of the moths. Linalool, eucalyptol, and high doses of geranyl acetate showed repellent effects on the codling moths, while myrcene, β-ocimene, nonanal, methyl salicylate, α-farnesene, and heptaldehyde showed the opposite. The relative levels of heptaldehyde, geranyl acetate, nonanal, and methyl salicylate were high in the fruits, which is intimately related to the localization of the walnut fruit by female *C. pomonella*. As these volatiles appear to be closely associated with the behavior of codling moths, we speculate that this work is useful for developing novel plant-derived attractants/repellents for these moths and, further, to understand the mechanism of recognition of walnut fruit in this species.

**Abstract:**

The volatile organic compounds (VOCs) of plant hosts allow insect localization through olfactory recognition. In this study, the oviposition behavior of the codling moth was investigated and the VOCs from different walnut organs were extracted and analyzed to systematically study their composition and content differences. The electrophysiological and behavioral responses of the codling moth to walnut VOCs were measured using gas chromatography–electroantennographic detection (GC-EAD) and a four-arm olfactometer to screen the key active contents. The field investigation results indicated that 90.3% of the eggs spawned by the first generation of adult codling moths were adjacent to the walnut fruits. Walnut VOCs are mainly composed of terpenes, aromatics, and alkanes. Twelve VOCs can produce electroantennogenic (EAG) responses in the codling moths. Both adult males and females exhibit concentration dependence, with notable disparities in their EAG response levels. In the olfactory behavioral bioassay, linalool, eucalyptol, and high doses of geranyl acetate showed repellent effects on the codling moths, while myrcene, β-ocimene, nonanal, methyl salicylate, α-farnesene, and heptaldehyde showed the opposite. The relative levels of heptaldehyde, geranyl acetate, nonanal, and methyl salicylate were high in the fruits, which is intimately related to the localization of the walnut fruit by females. These VOCs can influence the oviposition behavior of codling moths but their application in the control of this pest needs to be confirmed and improved through further field experiments.

## 1. Introduction

The codling moth, *Cydia pomonella* L. (Lepidoptera: Tortricidae), is an omnivorous pest of walnuts worldwide. Their larvae bore into and fill the fruits with feces, causing substantial economic loss [1]. Chemical pesticides are mainly used for codling moth prevention but the excessive use of pesticides can lead to resistance in codling moths, which causes a reduction in their effectiveness [2,3,4]. Insecticide-resistant populations of codling moths have a significantly increased response to plant volatile organic compounds (VOCs) [5], suggesting that these VOCs may provide more effective control in areas where insecticides have been extensively used.

In recent years, there has been increasing interest in the chemical ecological regulation of the codling moth. However, most studies have primarily focused on regulating the moth’s sex pheromones, with relatively little research on regulating the volatile compounds emitted by host plants. Although the use of sex pheromones to trap pests may reduce the population density of male insects, it is less effective on females, which are more closely related to the degree of damage to fruit trees; i.e., each female can lay up to 160–240 eggs on the fruits [6]. Hence, in order to decrease the population of pests and mitigate the damage caused by female insects, it is imperative to conduct additional research on attractants and repellents specifically designed to target adult female insects.

There are universal information channels in insects of the family Tortricidae to recognize pheromones in the environment [7], which regulate their behavior and physiological changes. Plant VOCs are a class of volatile secondary substances emitted from plant organs that promote attraction or avoidance of pests, which provide insights for the development of new ecological pesticides [8,9]. Plant VOCs are related to factors such as variety [10,11,12], rootstock [13], and soil [14]. They can serve as a medium for information exchange, playing an important role in the olfactory host localization of insects [15,16]. Many plant volatiles have been found to serve as attractants for insect oviposition, such as *Anopheles* mosquitoes (Diptera: Culicidae) [17], *Helicoverpa armigera* (Lepidoptera: Noctuidae) [18], and *Phthorimaea operculella* (Lepidoptera: Gelechiidae) [19].

The codling moth exhibits a robust response to various compounds found in volatiles from apples (Rosaceae: Malus) and pears (Rosaceae: Pyrus), including (*E*,*E*)-α-farnesene and butyl hexanoate. These are the main substances released from the headspace of apple trees and they are particularly attractive to female moths. Among these compounds, (*E*,*E*)-α-farnesene is an egg-laying stimulant for females and has a strong attractive effect on them. Butyl hexanoate is attractive to gravid females [20,21,22]. Moreover, (*E*,*Z*)-2,4-decadienoate (pear ester), the main volatiles in pear fruit, are as effective as sex pheromones in attracting codling moths [23]. A variety of compounds capable of EAG responses in codling moths have also been found within walnuts, *Juglans regia* L. (Juglandaceae: Juglans), such as nonanal and methyl salicylate [24,25].

The larvae of the codling moth tunnel into the fruit, causing harm. However, these larvae have limited crawling ability. As a result, the survival rate of the larvae is significantly impacted by the area where the female moth chooses to lay her eggs. Therefore, adult female moths primarily rely on olfactory senses to identify suitable locations for egg-laying [25]. It has been shown that females lay their eggs within 10–15 cm of the fruit [26,27], suggesting that certain volatile components in the fruit may have an attractive effect on the oviposition and larval feeding of codling moths. Previous research on the volatile compounds emitted by walnut trees has primarily examined entire clusters of branches [21,24,25,28]. There have been fewer studies comparing the volatiles emitted by individual organs such as leaves and fruits, as well as branches [29,30]. This lack of comparative studies makes it challenging to determine the similarities and differences in the volatile compounds emitted by different parts of the walnut tree, as well as the specific substances in the fruits that attract adult insects to lay eggs.

Walnut is a woody plant of the Juglandaceae. The prevalence of codling moths has been escalating in recent years, resulting in a significant decrease in fruit yield. This has led to substantial economic losses in the forestry and fruit industries, particularly in South Xinjiang. It is crucial to develop preventive measures to efficiently decrease the population of pests. In this study, we employed GC-MS to identify the primary volatile components present in various parts of walnuts. Specifically, we analyzed the leaves, fruits, and branches of the walnut tree. The obtained results were then compared with the GC-EAD results. Several compounds that can cause the antennal potential response of the codling moth were screened by EAG and olfactory behavior testing, clarifying the main volatiles that affect the oviposition selection of the codling moth for different walnut organs. These results are an important reference for the development of attractants or repellents for the codling moth that are based on host plant volatiles.

## 2. Methods and Materials

### 2.1. Insect Rearing

The test insects were collected from the Hotan area of Xinjiang (37°5′46″ N, 79°73′81″ E). The walnut orchard consists of three main varieties of ‘Xinfeng’, ‘Wen 185’ and ‘Zha 343’. The codling moth larvae were collected from the trunks of trees in walnut orchards and brought back to the laboratory for rearing and to wait for eclosion to be used for testing. The larvae were individually reared in Petri dishes to identify the males and females after pupation and 10 pairs of adults were reared in each cage with a side length of 25 cm. All of the insects were reared under the conditions of 25 ± 0.5 °C, 70% ± 5% relative humidity and a 16:8 h light/dark photoperiod with 600 lx light intensity. A 10% honey water solution was provided ad libitum.

### 2.2. Investigation of Oviposition Behavior

#### 2.2.1. Oviposition Organ Selection

The investigations in this study were conducted after the peak of adult incidence [31]. Five points were selected in the east, south, west, north, and center of the walnut orchard, and five walnut trees were selected, with each point repeated five times. Each cluster of walnut branches was collected according to four directions: east, south, west, and north, from the upper (over 8 m), middle (5–8 m), and lower (2–5 m) crowns of each tree. Each cluster contained 5–8 branches. After collection, the samples were brought back to the laboratory so that the position of the eggs could be observed with a stereomicroscope (SZM45-ST1, Shanghai Optical Instruments, Shanghai, China). The observations were repeated three times for each group of 100 eggs.

#### 2.2.2. Investigation of Egg-Laying Location

In addition, we investigated eggs laid on leaves and branches. Two hundred eggs that were not laid on fruit were randomly surveyed in the field. The number of fruits within a 20 cm radius of each egg was recorded.

### 2.3. Extraction and Identification of Volatiles

Healthy 20-year-old ‘Wen 185’ walnut trees were selected for the collection of volatiles from the different organs of the walnuts using headspace dynamic adsorption. For gas collection, one end of the odorless silica gel tube was connected to live walnut leaves, branches, and fruits, which were covered with polyethylene bags using an activated charcoal filter tube. The other end was connected to an adsorbent tube (made of stainless steel with an outer diameter of 6 mm and a length of 100 mm and filled with the adsorbent Tenax-TA) using an atmospheric sampling instrument (QC-1S, Labor Protection Science, Beijing, China). The flow rate was 0.8 L·min^−1^ and the sampling time was 6 h. The experiment was repeated 10 times. After collection, the samples were eluted with 1.5 mL of hexane and concentrated to 0.5 mL. The compounds in the eluate were identified using an Agilent 5977B GC/MSD GC-MS instrument (Agilent Technologies, Santa Clara, CA, USA). The column was an HP-5 measuring 30 m × 0.32 mm with a film thickness of 1.0 µm. The carrier gas flow rate was 1 mL min^−1^ with a split ratio of 5:1 and the transfer line temperature was 250 °C. The temperature increase was programmed from 50 °C (held for 5 min) at 8 °C·min^−1^ to 230 °C (held for 10 min). The mass spectrometry was performed at an ion source temperature of 280 °C, electron bombardment ionization (EI, 70 eV), full scan, scanning range of 30–400 m·z^−1^, and a resolution of 60,000. The data obtained from the assay were subjected to tracefinder deconvolution, peak extraction, and spectral library search (NIST, WILEY, and self-constructed libraries) for compositional identification. The compounds used as EAG tests and olfactory behavioral tests were verified by comparison with authentic standards, and other compounds by comparison with RI in the NIST database.

### 2.4. Test Conditions of Gas Chromatography-Electroantennographic Detection (GC-EAD)

Mated adults that were 3–5 days old were selected for testing. The tests were conducted during the peak daily egg-laying period of females from 19:00 to 21:00 [32]. The antennae of the adults were cut off from the base for subsequent assay tests with reference to a previous method [25]. The ends of the antennae were connected to a saline-filled glass capillary under an MP-15 micromanipulator (Tangshandinggan Technology, Tangshan, China) and the other side of the capillary was connected to a silver wire interfaced with an electroantennogram.

Recording equipment system: The eluent from walnut organs was split between the FID and the tentacle holder. One side joined the activated charcoal-filtered and humidified air stream (0.5 L·min^−1^) to a stainless-steel tube (10 mm ID) with the antennae 0.5 cm from the end of the tube and the other half entered the GC under the same conditions as in Section 2.3. The EAD outlet was maintained at a temperature of 180 °C and the shunt ratio between the FID and EAD was 1:1. An IDAC-2 Bluetooth data-logging controller and IDAC-4 data-logging controller were used to record the data during the experiment (Syntech, Hilversum, The Netherlands), which were then analyzed using software GC-EAD 4.7 (Syntech, Kirchzarten, Germany).

### 2.5. Electroantennography (EAG)

EAG tests were performed with eight compounds from walnuts that elicited the electrophysiological responses of codling moths in the GC-EAD test. In addition, butyl hexanoate and (*E*,*Z*)-2,4-decadienoate (pear esters), the main volatile components of apples and pears, were added. α-arnesene (purity ≥ 98%), myrcene (purity ≥ 90%), eucalyptol (purity 99%), β-ocimene (purity ≥ 90%), linalool (purity 98%), nonanal (purity 96%), methyl salicylate (purity 99%), heptaldehyde (purity 97%), geranyl acetate (purity 96%), butyl hexanoate (purity ≥ 98%), and pear esters (purity ≥ 97%) were purchased from Macklin Reagent (Shanghai, China). Hexane, purity 98%, was used to dilute the volatiles, which were stored at −20 °C or 2–8 °C until use.

The test time was the same as that described in Section 2.4. The test followed the previously described method with modifications [33]. A 10 μL sample of different concentrations of volatile substances diluted with hexane was withdrawn and added dropwise onto a piece of filter paper (20 mm × 8 mm). After 20 s, the filter paper was placed into a Pasteur tube with tweezers and both ends of the tube were sealed with sealing film. The sample was volatilized for 1 min and then measured. The antennae were stimulated with a stimulation calendar period of 500 ms and a stimulation interval of about 30 s. The flow rate of the continuous airflow was the same as that of the stimulating airflow at 4 mL s^−1^. Only 1 antenna was taken from each adult codling moth; 6 antennae were tested in each treatment, and each antenna test was replicated 3 times. Hexane was used as a blank control to reduce the error before and after each test.

### 2.6. Olfactory Behavior Bioassays

The volatile compounds in 2.5 were selected for the study of adult behavioral responses using a four-arm olfactometer (Hefan Technology, Shanghai, China) at the same time. The test methodology was based on previous work with modifications [34,35]. Groups of 50 insects for each odor compound, male and female, were tested 10 at a time, and their selection behavior was determined over 5 min. The test was repeated five times. Before the test, the four-arm olfactometer parts were cleaned with degreasing cotton wool and alcohol, dried, and deodorized for use. The four-arm olfactometer was connected to the vacuum pump and other accessories with silicone tubing and test-run for 3 min to ensure that the overall airtightness was satisfactory. The volatiles were diluted and 10 μL was added evenly onto a circular filter paper with a diameter of 3 cm (the active ingredients were 0, 10, 100, and 1000 μg). The filter paper was folded and placed in the flavor bottle. For each experiment, aliquots (10 μL) of each of the 3 concentrations of each compound, adsorbed onto a filter, and the fourth arm was used as a control, with a 10 uL drop of paraffin liquid on an equal sized filter paper. A stream of purified moist air was delivered at a constant rate of 0.5 L·min^−1^. All tests were carried out at a room temperature of 25 ± 2 °C. The four-arm olfactometer was turned 90° in the direction of each set of measurements to avoid mechanical errors.

### 2.7. Statistical Analysis

All analyses of the results were carried out using IBM SPSS 22.0 and variances were analyzed using a one-way ANOVA test for oviposition organ selection, *t*-tests, and one-way ANOVA tests for variances in EAG responses, and Wilcoxon rank sum tests to determine differences between the means in the four-arm olfactometer bioassays.

## 3. Results

### 3.1. Oviposition Behavior

**Figure 1:**
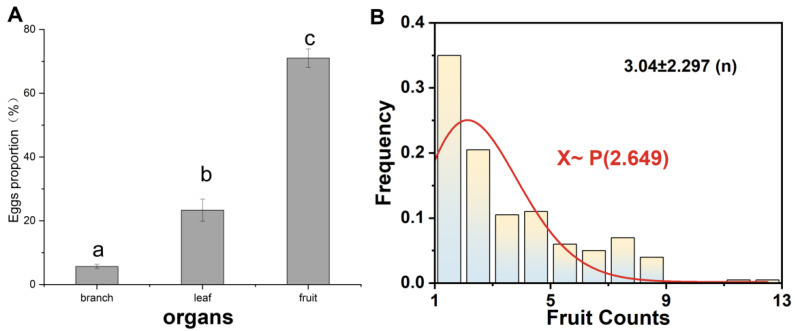
(**A**) Proportion of eggs on each organ of walnut. Different letters represent the presence of significant differences (ANOVA, LSD test). (**B**) Histogram of frequency distribution of fruit counts.

The first generation of codling moths favored laying their eggs on the fruit (Figure 1) and there was a significant difference (*p* < 0.01) in egg-laying among the three different organs. In addition, 90.3% of the eggs were found within 20 cm of the fruit. The mean distance of the eggs from the fruit was 10.94 ± 5.64 cm. Eggs in the range of 1–13 fruits conformed to a Poisson distribution. The expected value of the number of fruits was 3.04, which indicated a range of 3–4 fruits in the vicinity of each egg, which was the ideal location for the female adults to lay their eggs.

### 3.2. Chemical Identification of Volatiles

A total of 54 volatile compounds were detected in 3 different walnut organs (Table 1), mainly including terpenes, aromatics, and alkanes. Among these, terpenes are more diverse and abundant and are important compounds that contribute to the odor of walnuts. The leaves included a greater variety of volatiles, consisting of 39 different species, while the fruits had a smaller number of volatiles, including just 28 species. The only volatiles detected in the fruit were heptaldehyde, 2,4-dimethylheptane, 2-methylnaphthalene, geranyl acetate, dodecane, 3-methyl-, and 4,4-dimethyl-1-hexene. The compounds whose relative content in the fruit was more than twice as high as in other organs were methylcyclohexane, carvone, toluene, styrene, 2,6,10-Trimethyl-dodecan, nonanal, methyl salicylate, decane, 2,9-dimethyl-, tridecane, and dibutyl phthalate.

### 3.3. Identification of the Candidate Volatiles

**Figure 2:**
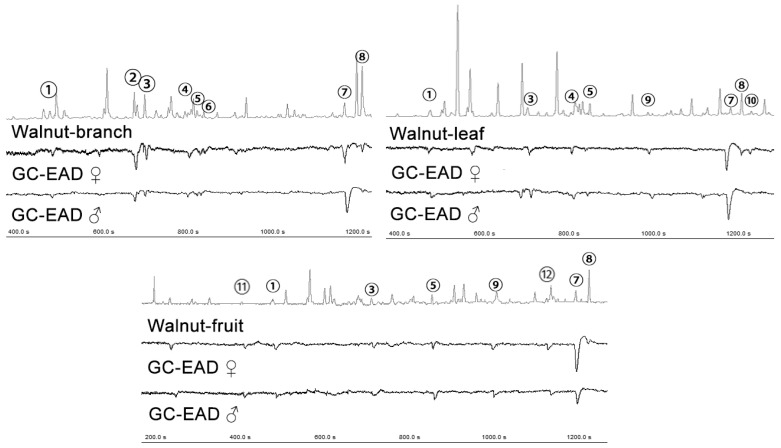
Gas chromatography–electroantennogram detection of codling moth. ① Myrcene, ② eucalyptol, ③ β-ocimene, ④ linalool, ⑤ nonanal, ⑥ (3*E*)-4,8-dimethylnona-1,3,7-triene, ⑦ (*E*) β-farnesene, ⑧ germacrene D, ⑨ methyl salicylate, ⑩ α-farnesene, ⑪ heptaldehyde, ⑫ geranyl acetate.

A total of 12 volatiles elicited antennae responses in female adults. The identity of these compounds was confirmed by comparing their retention times and mass spectra with reference compounds (Figure 2). Among them, myrcene, eucalyptol, β-ocimene, linalool, nonanal, germacrene D, and (*E*)-β-farnesene were detected in the walnut fruits, leaves, and branches. Heptaldehyde and geranyl acetate were detected in the fruits and α-farnesene was detected exclusively in the leaves. (3E)-4,8-dimethylnona-1,3,7-triene was detected in the leaves and branches but was not detected in the fruits. Methyl salicylate was detected in the leaves and fruits, but not in the branches.

### 3.4. EAG Response to Candidate Volatiles

**Figure 3:**
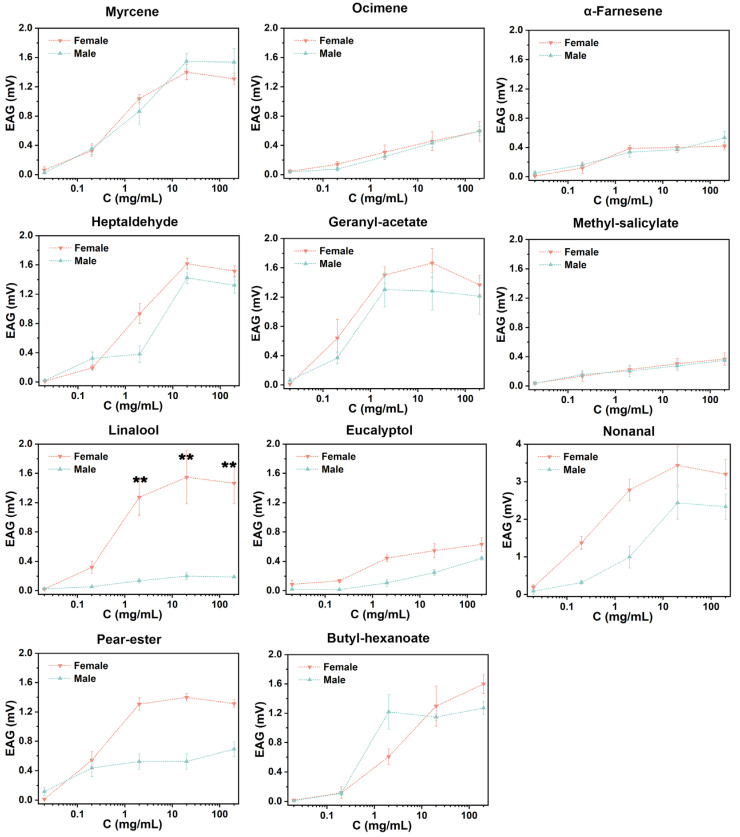
Electrophysiologic responses of antennae elicited by different doses of volatiles. ** indicates a significant difference between different sexes for the same concentration. (*t*-test, *p* < 0.01).

**Figure 4:**
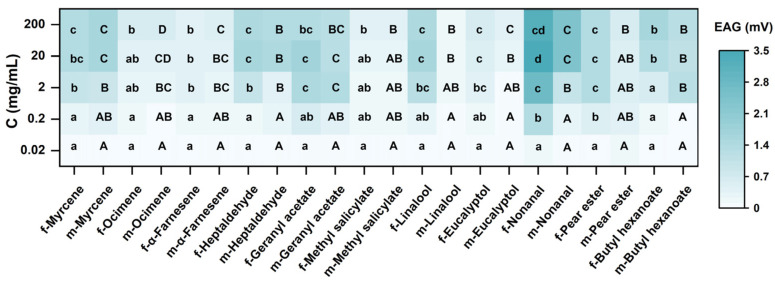
EAG response from different doses of volatiles. Different lowercase letters indicate significant differences between females and different uppercase letters indicate significant differences between males; f means females and m means males. (*t*-test, *p* < 0.05).

All compounds were significantly different from the control at the highest response value (*t*-test, *p* < 0.05). Olfactory differentiation was not obvious in adults of different sexes (Figure 3). Highly significant differences were found exclusively for linalool (2 mg·mL^−1^, 20 mg·mL^−1^, 200 mg·mL^−1^) (*t*-test, *p* < 0.05). The females responded more intensely to nonanal, geranyl acetate, and linalool, with the maximum EAG response value of 3.43 ± 0.23 mV at a concentration of 20 mg·mL^−1^ for nonanal. However, the males responded more intensely to nonanal, which reached a value of 2.44 ± 0.19 mV at a concentration of 20 mg·mL^−1^ (Figure 4). Adult codling moths were sensitive to the concentrations of the compounds and there were significant differences in the responses to different concentrations of each compound. These findings revealed that the olfactory sensation of codling moths is closely related to the concentration of the compounds in nature (Table 2).

### 3.5. Olfactory Behavior

The olfactory behavior of the codling moths reflected a relatively similar trend to the EAG response, with 100 mg of nonanal being more effective in attracting female adults (19.80 ± 1.39) and 1000 mg of methyl salicylate being more effective in attracting male adults (20.80 ± 2.13). Eucalyptol and linalool act as repellents to codling moths. The mean values of the selection of both repellant volatiles were higher in females than males, in addition to the other compounds that behaved as attractants. Geranyl acetate attracts codling moths at low concentrations, while it has a repellent effect on codling moths at high concentrations. The selection of volatiles by the codling moth females and males was broadly similar, with significant differences only found in the selection of heptaldehyde active ingredients at 100 mg and 1000 mg, linalool at 0 mg and 10 mg, eucalyptol at 0 mg, and pear ester at 10 mg. In addition, concentration had a strong effect on selection by codling moths, with significant differences caused by differences in the content of the active ingredient in each of the eleven volatiles tested (Table 2). Codling moths adults were infrequently active and 37.73 ± 14.33% of the females and 33.2 ± 16.34% of the males did not make a choice in this test.

## 4. Discussion

The codling moth adult has degenerated compound eyes and prefers to be active at night, so olfaction plays a key role in the host localization by this insect. Both physical and chemical factors influence the oviposition behavior of adult females, such as similarities and differences in host plant volatiles and the friction of the oviposition substrate [25,36,37]. In the walnut orchards observed in this study, the eggs were mostly laid on the fruit, which is consistent with the results of previous investigations in other orchards [38]. In this study, the selected orchard was located in the region of Hotan, Xinjiang, which has an arid climate with sparse rainfall. However, the region is prone to sandy and dusty weather and a large amount of sandy soil adheres to the leaves and near the base of the fruit stalks, which may affect the egg-laying of codling moths. Female adults tend to lay their eggs closer to the fruit, which may be related to the higher content of nonanal, methyl salicylate, heptaldehyde, and geranyl acetate in the volatiles of the fruit.

Walnut fruits contain high relative levels of nonanal and methyl salicylate, which have been shown to elicit the EAG response in codling moths in previous studies [25] and also showed strong attraction to females according to the olfactory behavior bioassay in this study. Nonanal is attractive to many Lepidoptera [39,40], which also specifically attracts females of *Helicoverpa assulta* (Lepidoptera: Noctuidae) and *Grapholita molesta* (Lepidoptera: Tortricidae) [41,42] and enables codling moth larvae to aggregate [43]. Methyl salicylate is widespread in many plants and is a metabolite associated with the salicylic acid pathway. Salicylic acid and methyl salicylate can transform into each other under certain conditions and play the role of protecting against unsuitable external conditions [44]. Several studies showed an increase in the content of methyl salicylate after plant injury [45,46]. Methyl salicylate can attract females of *Argyresthia conjugella* (Lepidoptera: Yponomeutidae) [47] and *Agriotes brevis* (Coleoptera: Elateridae) [48] and it can induce females of *Spodoptera frugiperda* (Lepidoptera: Noctuidae) to lay eggs [46], likewise serving to attract their predators [49,50]. Codling moths tend to aggregate [51,52]. Therefore, a high concentration of methyl salicylate may release a signal that the plant has been damaged and guide the codling moth to aggregate. Although methyl salicylate elicited a low EAG response, it attracted more adults in the olfactory behavior bioassay, which may be related to the diffusion rate of the substance.

**Figure 5:**
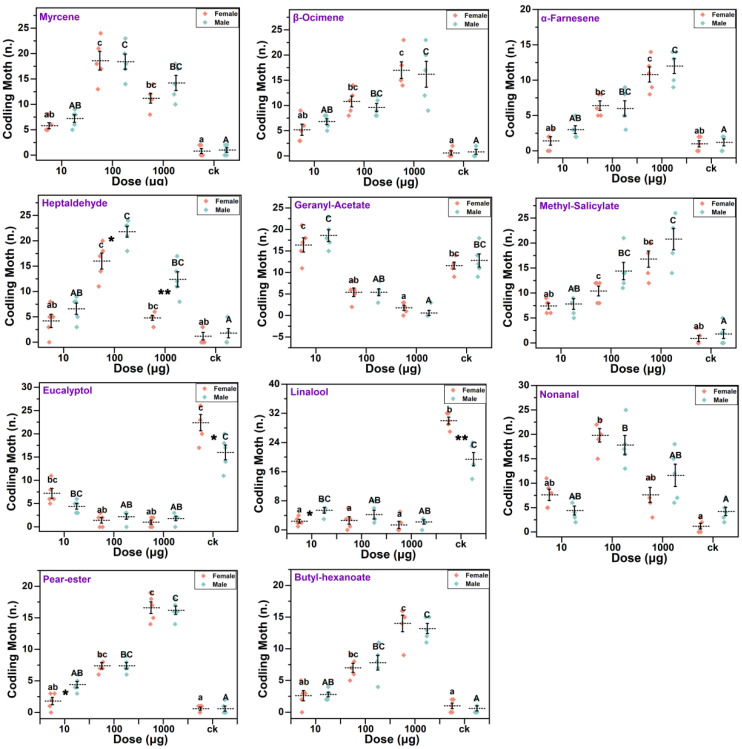
Olfactory response to different doses of compounds. Different lowercase letters indicate significant differences between females, different uppercase letters indicate significant differences between males (Wilcoxon test, *p* < 0.05), * represents significant differences between males and females (*t*-test, *p* < 0.05), and ** represents highly significant differences between males and females (*t*-test, *p* < 0.01).

Among the three organs, geranyl acetate and heptaldehyde were detected exclusively in the fruit. Geranyl acetate is a natural monoterpene with excellent bacteriostatic and antioxidant activities [53,54] and it is repellent to many insects [55]. However, it can elicit an EAG response in *S. frugiperda* [56] and, under certain conditions, it can also induce females to lay eggs [46]. In the present study, geranyl acetate, which was detected exclusively in fruits, elicited a robust EAG response and attracted adult codling moths even at low concentrations. This suggests an association between the searching behavior of codling moths for mating sites and the levels of geranyl acetate in their surroundings. Heptaldehyde is mainly found in nuts and volatile oils and is a substance that makes up the flavor of ripe nuts [57,58,59]. It has been reported to elicit EAG responses in parasitic wasps, a predator of many lepidopteran pests [60,61,62]. In this study, it was revealed that the insecticide induced a relatively intensive EAG response in codling moths and attracted both male and female adults within a certain dosage range. Concentration dependence was evident for all volatiles in both male and female adults. Significant differences in EAG response and olfactory behavioral responses were obtained for different concentrations (Figure 4 and Figure 5). The oviposition behavior of the codling moth may be associated with the contents of nonanal, methyl salicylate, heptaldehyde, and geranyl acetate in the fruits.

In this study, butyl hexanoate and pear ester, which are good attractants for codling moths derived from apples and pears in previous studies, were used as controls [20,21,22]. Our results revealed that myrcene, β-ocimene, and methyl salicylate can achieve comparable attractant effects to pear ester in olfactory behavioral bioassays, which indicated that these three volatiles have the potential to contribute to the development of traps for female adults in the future.

Linalool is a chained terpene alcohol that kills or repels many insects [63,64] and it also prevents egg-laying by insects such as *Ceratitis capitata* (Diptera: Tephritidae) and *Plutella xylostella* (Lepidoptera: Plutellidae) [65,66]. In this study, it was also observed that high doses of linalool elicited significantly higher EAG intensities in females than in males (Figure 3). In the presence of linalool stimuli in the olfactory test, the females were more repelled than the males (Figure 5). This indicates that linalool has a good repellent effect on adult female codling moths, which may be valuable in the future development of insect-repellent agents for females.

Both female and male codling moths exhibited comparable responses to plant volatiles, indicating that there is no apparent distinction in terms of olfactory perception based on sex. This suggests that sex pheromones may not be the only way by which males locate females. In the present study, it was found that female adults were less disturbed by external factors. Even during the most active period of the experiment, there were still many females who did not respond to the olfactory behavior test; in Figure 5, we reached similar conclusions to previous studies [67].

Previous traps based on sex pheromones were only able to trap male adults. In this study, we found that some plant-derived odors can be a better attractor for both sexes. The major limitation of our study is mainly laboratory research. Further research should be undertaken with field trials to verify the attraction or repellent effects of these compounds in the field. In addition, previous research has been conducted on the chemosensory mechanism of codling moths and there is already a relevant understanding of its pheromone-sensing mechanism and olfactory proteins within the antennae. Many studies have focused on insect populations with apples and pears as hosts, specifying receptor genes for volatiles such as pear esters and sex pheromones [68,69,70]. Recognition genes related to walnut volatiles have not yet been studied and there is an urgent need for this next step in the discovery of related olfactory genes to gain a deeper understanding of the recognition mechanisms between codling moths and plant volatiles.

## 5. Conclusions

Adult females may delay their departure after choosing an optimal spot for mating and laying eggs, while males may use plant odors to find females and identify a more favorable location. The codling moth always lays its eggs around or directly on the fruit, relative levels of heptaldehyde, geranyl acetate, nonanal, and methyl salicylate were high in the fruits, and all of these compounds act as attractants for codling moths, which indicates that these compounds are closely related to the localization of walnut fruits by females.

## Figures and Tables

**Table 1:** Volatile components in various organs of walnut.

No.	RI	Chemical Formula	CAS No.	Compound	Relative Content (%)		
					Leaf	Fruit	Branch
1	722	C_7_H_14_	108-87-2	Methylcyclohexane	0.43	1.31	nd
2	786	C_7_H_8_	108-88-3	Toluene	0.78	2.95	nd
3	802	C_8_H_18_	111-65-9	Octane	nd	nd	11.01
4	824	C_9_H_20_	2213-23-2	2,4-Dimethylheptane	nd	2.54	nd
5	876	C_8_H_18_O	142-96-1	Butyl ether	0.72	nd	nd
6	878	C_8_H_8_	100-42-5	Styrene	0.46	1.58	nd
7	881	C_7_H_14_O	111-71-7	Heptaldehyde	nd	1.26	nd
8	936	C_10_H_16_	80-56-8	α-Pinene	0.41	nd	nd
9	974	C_10_H_16_	127-91-3	β-Pinene	3.55	3.2	4.13
10	989	C_10_H_16_	123-35-3	Myrcene	1.15	1.68	0.9
11	992	C_8_H_14_O_2_	3681-71-8	Hexenyl acetate	25.41	nd	nd
12	994	C_12_H_26_	13475-82-6	2,2,4,6,6-Pentamethyl-heptane	0.44	nd	0.68
13	997	C_10_H_16_	99-83-2	α-Phellandrene	0.63	nd	0.51
14	1015	C_10_H_22_	124-18-5	Decane	nd	nd	1.7
15	1020	C_10_H_16_	555-10-2	β-Phellandrene	0.69	nd	1.44
16	1024	C_10_H_16_	5989-27-5	Limonene	10.54	8.92	10.66
17	1032	C_10_H_18_O	470-82-6	Eucalyptol	0.12	0.11	0.66
18	1039	C_10_H_16_	13877-91-3	β-Ocimene	5.85	3.75	1.75
19	1080	C_10_H_14_	99-87-6	p-Cymene	2.12	2.09	2.45
20	1086	C_10_H_18_O	78-70-6	Linalool	1.09	1.43	4.15
21	1099	C_9_H_18_O	124-19-6	Nonanal	1.2	5.81	1.79
22	1123	C_11_H_18_	19945-61-0	(3*E*)-4,8-Dimethylnona-1,3,7-triene	8.58	nd	3.66
23	1131	C_10_H_16_O	1753-35-1	3(10)-Caren-4-ol	nd	nd	0.66
24	1135	C_10_H_16_O	473-67-6	Berbenol	2.12	nd	nd
25	1138	C_12_H_26_	1002-17-1	2,9-Dimethyl-decane	0.54	2.76	nd
26	1141	C_10_H_14_O	30460-92-5	Pinocarvon	0.78	nd	0.54
27	1160	C_10_H_20_O	2216-51-5	L-Menthol	0.35	nd	1.42
28	1162	C_10_H_20_O	98167-53-4	(−)-Menthyl alcohol	0.6	nd	nd
29	1163	C_10_H_14_O	1197-01-9	p-Cymen-8-ol	0.95	nd	nd
30	1173	C_12_H_26_	1002-43-3	3-Methylundecane	0.24	nd	1.22
31	1175	C_8_H_8_O_3_	119-36-8	Methyl salicylate	1.48	6.53	nd
32	1176	C_10_H_18_O	98-55-5	α-Terpineol	nd	nd	1.09
33	1177	C_10_H_16_O	6712-79-4	Isopinocarveol	1.35	nd	nd
34	1215	C_12_H_26_	112-40-3	Dodecane	3.76	5.11	nd
35	1218	C_10_H_14_O	99-49-0	Carvone	0.49	6.7	0.86
36	1264	C_15_H_32_	31295-56-4	2,6,11-Trimethydodecane	0.37	nd	nd
37	1279	C_11_H_10_	91-57-6	2-Methylnaphthalene	nd	4.39	nd
38	1312	C_13_H_28_	629-50-5	Tridecane	4.29	14.28	4.47
39	1330	C_12_H_24_	294-62-2	Cyclododecan	2.72	nd	nd
40	1339	C_14_H_22_	61227-89-2	5,7-Diethyl-5,6-decadien-3-yne	1.68	nd	1.1
41	1343	C_12_H_20_O_2_	141-12-8	Neryl acetate	nd	nd	0.57
42	1361	C_12_H_20_O_2_	105-87-3	Geranyl acetate	nd	2.63	nd
43	1377	C_15_H_32_	3891-98-3	2,6,10-Trimethyl-dodecane	0.77	4.03	nd
44	1412	C_12_H_10_	827-54-3	2-Vinylnaphthalene	1.67	nd	nd
45	1413	C_14_H_30_	629-59-4	Tetradecane	1.71	1.21	1.73
46	1425	C_10_H_16_	36144-40-8	1-Butenylidene-cyclohexane	nd	nd	2.38
47	1448	C_15_H_24_	18794-84-8	(*E*)-β-Farnesene	1.38	2.79	2.18
48	1475	C_15_H_22_	644-30-4	α-Curcumene	nd	nd	1.56
49	1479	C_15_H_24_	23986-74-5	Germacrene D	7.49	2.03	15.48
50	1491	C_15_H_24_O	128-37-0	Butylated hydroxytoluene	nd	2.51	18.54
51	1495	C_15_H_24_	502-61-4	α-Farnesene	0.62	nd	nd
52	1555	C_13_H_28_	17312-57-1	3-Methyl-dodecane	nd	3.22	nd
53	1866	C_8_H_16_	1647-08-1	4,4-Dimethyl-1-hexene	nd	1.46	nd
54	1923	C_16_H_22_O_4_	84-74-2	Dibutyl phthalate	0.48	3.72	0.71

nd means not detected.

**Table 2:** ANOVA results of EAG and olfactory response tests with different concentrations of several compounds.

Compound		EAG			Olfactory Response		
		*F*	df	*sig.*	*chi-square*	df	*p*
myrcene	female	66.960	4	<0.01	15.000	3	0.002
	male	29.553	4	<0.01	14.755	3	0.002
β-ocimene	female	5.362	4	<0.05	15.000	3	0.002
	male	30.092	4	<0.01	13.653	3	0.003
α-farnesene	female	15.576	4	<0.01	13.776	3	0.003
	male	11.087	4	<0.01	13.250	3	0.004
heptaldehyde	female	87.935	4	<0.01	11.816	3	0.008
	male	52.706	4	<0.01	14.040	3	0.003
geranyl acetate	female	17.749	4	<0.01	13.080	3	0.004
	male	9.039	4	<0.01	14.755	3	0.002
methyl salicylate	female	4.503	4	<0.05	13.562	3	0.004
	male	5.178	4	<0.05	13.653	3	0.003
linalool	female	8.956	4	<0.01	10.340	3	0.016
	male	9.766	4	<0.01	10.714	3	0.013
eucalyptol	female	12.414	4	<0.01	14.125	3	0.003
	male	34.361	4	<0.01	13.174	3	0.004
nonanal	female	86.478	4	<0.01	13.560	3	0.004
	male	76.42	4	<0.01	12.437	3	0.006
pear ester	female	73.539	4	<0.01	14.040	3	0.003
	male	4.546	4	<0.05	15.000	3	0.002
butyl hexanoate	female	22.638	4	<0.01	14.040	3	0.003
	male	29.471	4	<0.01	15.000	3	0.002

## Data Availability

The original contributions presented in the study are included in the article material, further inquiries can be directed to the corresponding authors.
